# Conservation of a Neutralization Epitope of Human T-cell Leukemia Virus Type 1 (HTLV-1) among Currently Endemic Clinical Isolates in Okinawa, Japan

**DOI:** 10.3390/pathogens9020082

**Published:** 2020-01-27

**Authors:** Mariko Mizuguchi, Yoshiaki Takahashi, Reiko Tanaka, Takuya Fukushima, Yuetsu Tanaka

**Affiliations:** 1 Department of Immunology, Graduate School of Medicine, University of the Ryukyus, 207 Uehara, Nishihara-cho, Okinawa 903-0215, Japan; ytakah3@med.u-ryukyu.ac.jp (Y.T.); reiko_tanaka@s5.dion.ne.jp (R.T.); 2 Laboratory of Hematoimmunology, School of Health Sciences, Faculty of Medicine, University of the Ryukyus, 207 Uehara, Nishihara-cho, Okinawa 903-0215, Japan; fukutaku@med.u-ryukyu.ac.jp

**Keywords:** human T-cell leukemia virus type 1 (HTLV-1), adult T-cell leukemia/lymphoma (ATL), HTLV-1 envelope gp46, neutralization epitope

## Abstract

Approximately one-tenth of the 10 million individuals living with human T-cell leukemia virus type-1 (HTLV-1) worldwide live in Japan. Most of these infected individuals live in the southwest region of Japan, including Okinawa prefecture; however, currently no prophylactic vaccine against HTLV-1 infection is available. For preventing the HTLV-1 spread, we previously generated a humanized monoclonal antibody (hu-LAT-27) that mediates both neutralization and antibody-dependent cellular cytotoxicity (ADCC). The neutralization epitope of LAT-27 is a linear amino acid sequence from residue 191 to 196 (Leu-Pro-His-Ser-Asn-Leu) of the HTLV-1 envelope gp46 protein. Here, we found that the LAT-27 epitope is well conserved among HTLV-1 clinical isolates prevalent in Okinawa. The hu-LAT-27 treatment inhibited syncytium formation by these clinical HTLV-1 isolates. Although an amino acid substitution at residue 192 in the LAT-27 epitope from proline to serine was found in a few HTLV-1 isolates, hu-LAT-27 could still react with a synthetic peptide carrying this amino acid substitution. These findings demonstrate the wide spectrum of hu-LAT-27 reactivity, suggesting that hu-LAT-27 may be a candidate drug for prophylactic passive immunization against HTLV-1 infection.

## 1. Introduction

The number of human T-cell leukemia virus type 1 (HTLV-1)-infected individuals worldwide is estimated to be around 10 million [1]. HTLV-1 causes neoplastic and inflammatory diseases, such as adult T-cell leukemia/lymphoma (ATL) and HTLV-1-associated myelopathy/tropical spastic paraparesis (HAM/TSP), respectively [2,3,4,5]. HTLV-1 is transmitted through breastfeeding and sexual contact via the influx of bodily fluids containing infected cells. However, no prophylactic vaccine or drug against HTLV-1 infection has been developed to date. As humanized or human monoclonal antibodies (mAb) have been demonstrated to be safe and effective in various areas of medicine, passive immunization with an HTLV-1 neutralizing mAb has been suggested as a potentially effective strategy for preventing the spread of HTLV-1 [6,7,8,9,10].

The HTLV-1 envelope (env) spike consists of two glycoproteins, cell surface gp46 and transmembrane gp21, and is made up of a trimer of heterodimers [11]. On target cells, gp46 binds to cell surface receptors, such as glucose transporter type 1 (GLUT1), neuropilin 1 (NRP-1), and heparan sulfate proteoglycan (HSPG), allowing for virus entry in association with gp21 [12,13,14]. Several neutralization epitopes have been identified on gp46, with the major epitopes located in a region between amino acids 187 and 199 [15,16,17,18,19]. We previously developed an anti-gp46 neutralizing mAb of rat origin, LAT-27, which recognizes the gp46 amino acid sequences from position 191 to 196 (Leu-Pro-His-Ser-Asn-Leu) [19,20]. LAT-27 prevented the in vitro immortalization of normal peripheral blood mononuclear cells (PBMCs) by HTLV-1 and the cellular entry of HTLV-1 env-expressing vesicular stomatitis virus (VSV) pseudotypes to target cells [19,21]. LAT-27 also protected newborn WKA rats from HTLV-1 infection [20]. Moreover, LAT-27 shows potential for eliciting antibody-dependent cellular cytotoxicity (ADCC) along with natural killer (NK) cells by binding to the proline-rich hinge domain of gp46, and eliminated HTLV-1-infected cells in vitro [22]. For its clinical application, LAT-27 has been humanized using gene recombination techniques [20]. This mAb (hu-LAT-27) was reported to block horizontal HTLV-1 infection in animal models [20], suggesting that hu-LAT-27 may be a candidate passive immunization drug against infection with HTLV-1.

Nevertheless, validating the efficacy of hu-LAT-27 requires extensive studies, including the determination of whether the LAT-27 epitope is well conserved in clinical HTLV-1 strains. Certain HTLV-1 stains isolated in the Caribbean, French Guiana, Gabon, and Brazil carry an amino acid substitution of proline to serine at residue 192 (P192S) in the LAT-27 epitope region [23,24,25]. In the present study, we demonstrate that the LAT-27 epitope is widely conserved among clinical HTLV-1 isolates prevalent in Okinawa prefecture, one of the most HTLV-1-endemic areas in Japan, and that hu-LAT-27 was able to neutralize all the HTLV-1 strains tested. In addition, hu-LAT-27 reacted with a synthetic peptide containing the P192S substitution. Thus, our findings suggest the potential of hu-LAT-27 in preventing the transmission of the currently prevalent HTLV-1 strains.

## 2. Results

### 2.1. The Major Neutralizing Domain of gp46 is Conserved in Clinical Isolates of HTLV-1

**Figure 1:**
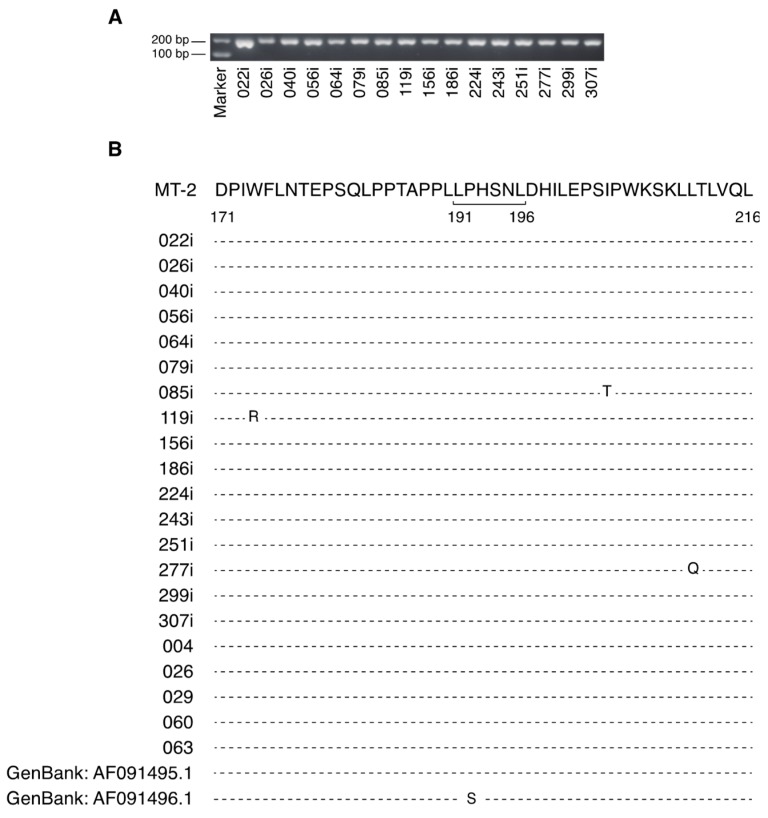
Conservation of a neutralizing domain of gp46 in clinical HTLV-1 isolates. (**A**) The 187-base-pair region of gp46 from clinical HTLV-1 isolates was amplified and subjected to gel electrophoresis. (**B**) Reference amino acid sequences of the MT-2 strain. The LAT-27 epitope is shown from residue 191 to 196 in the sequence. Changes in amino acids are indicated along the standard line aligned with the MT-2 sequences.

LAT-27 inhibits HTLV-1 transmission by binding to the neutralization epitope spanning the gp46 amino acid sequence from residue 191 to 196 [19]. A P192S amino acid substitution has been found in the LAT-27 epitope region (Figure 1B). Therefore, to determine whether the neutralization domain is conserved among various clinical isolates, HTLV-1 genomic sequences were analyzed using 16 newly established HTLV-1-infected cell lines originating from various subtypes of patients with ATL living in Okinawa (Table 1). First, the deletion or insertion of the HTLV-1 genomic region from nucleotide 5691 to 5877, containing the major neutralizing domain of gp46, was evaluated by PCR amplification. In all the HTLV-1 isolates tested, 187-base-pair DNA fragments of the gp46 region were detected (Figure 1A). Subsequently, the gp46 amino acid sequence from residue 171 to 216 in each clinical HTLV-1 isolate was examined and compared to the reference HTLV-1 sequences of strain MT-2 [26]. Among the 46 amino acids of the gp46 region, the tested HTLV-1 isolates showed little or no change in the amino acid sequence compared to that of the reference strain (Figure 1B). Although a change of one nucleotide from C to T at position 5778 within the LAT-27 epitope was observed in strain 224i (data not shown), no amino acid substitution was found in the neutralization epitope (Figure 1B). 

The present experimental conditions may have limited the possibility of detecting intra-host polymorphisms of HTLV-1 since the LAT-27 epitope region was analyzed using established cell lines. To address this issue, we further examined this region of the LAT-27 epitope using genomic DNA from fresh PBMCs from acute ATL patients (#004, #026, #029, #060, and #063), and found no intra-host polymorphism (Figure 1B). These results indicate that the major neutralizing domain of gp46 is well conserved in the HTLV-1 strains currently prevalent in Okinawa.

### 2.2. hu-LAT-27 Inhibits In Vitro Infection with Clinical HTLV-1 Isolates

**Figure 2:**
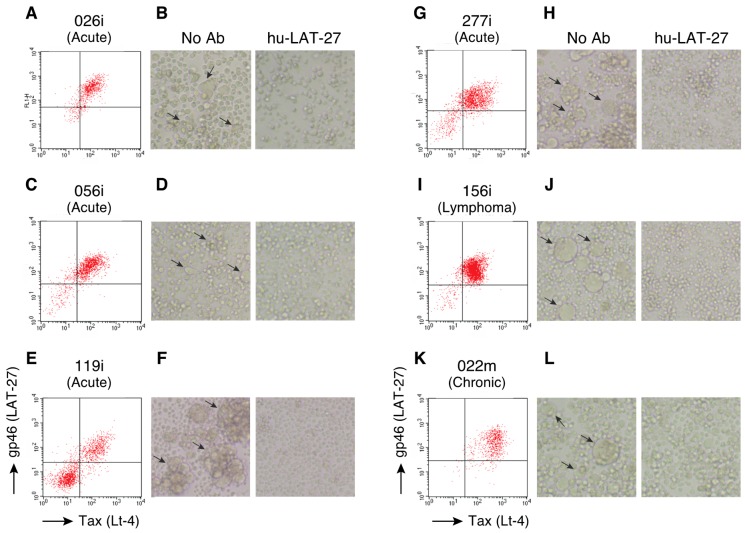
Neutralization of clinical HTLV-1 isolates by hu-LAT-27. (**A**, **C**, **E**, **G**, **I**, **K**) HTLV-1-infected cell lines, 026i, 056i, 119i, 277i, 156i, and 022m, were cultured with PGE2 for 24 h. The cells were then harvested and stained with FITC-conjugated anti-gp46 antibody (LAT-27) and HyLite Fluor 647-labeled anti-Tax antibody (Lt-4), and then subjected to FCM. (**B**, **D**, **F**, **H**, **J**, **L**) PGE2-treated HTLV-1-infected cells were co-cultured with CEM cells in the presence or absence of hu-LAT-27 for 24 h. Syncytium formation was microscopically observed using an inverted microscope at a magnification of 100×. The arrows indicate syncytium formation. Experiments were independently repeated three times.

The expression of the LAT-27 epitope in HTLV-1-infected cell lines was further examined using flow cytometry (FCM). HTLV-1-infected cell lines were treated with prostaglandin E2 (PGE2) to induce the maximum expression of HTLV-1 antigens, including gp46 and Tax [27]. High levels of LAT-27 epitope expression were observed in the HTLV-1-infected cell lines (Figure 2A, C, E, G, I, and K). There were two populations, gp46- and Tax-negative and positive cells, among the 119i cells (Figure 2E). It remains to be determined whether these gp46- and Tax-negative cells did not express viral proteins because of the mutations of the provirus. 

To confirm the neutralizing potential of hu-LAT-27 against these clinical HTLV-1 isolates, we performed a syncytium inhibition assay. PGE2-treated-HTLV-1-infected cell lines and an HTLV-1-uninfected target cell line, CEM, were co-cultured with or without hu-LAT-27 for 24 h. Syncytium formation was completely inhibited by the addition of hu-LAT-27 (Figure 2B, D, F, H, J, and L), which was observed for up to 3 days (data not shown). These results demonstrate that hu-LAT-27 has a broad neutralizing spectrum against the clinical HTLV-1 isolates currently prevalent in Okinawa.

### 2.3. The LAT-27 Epitope is Conserved in HTLV-1-Infected Individuals

**Figure 3:**
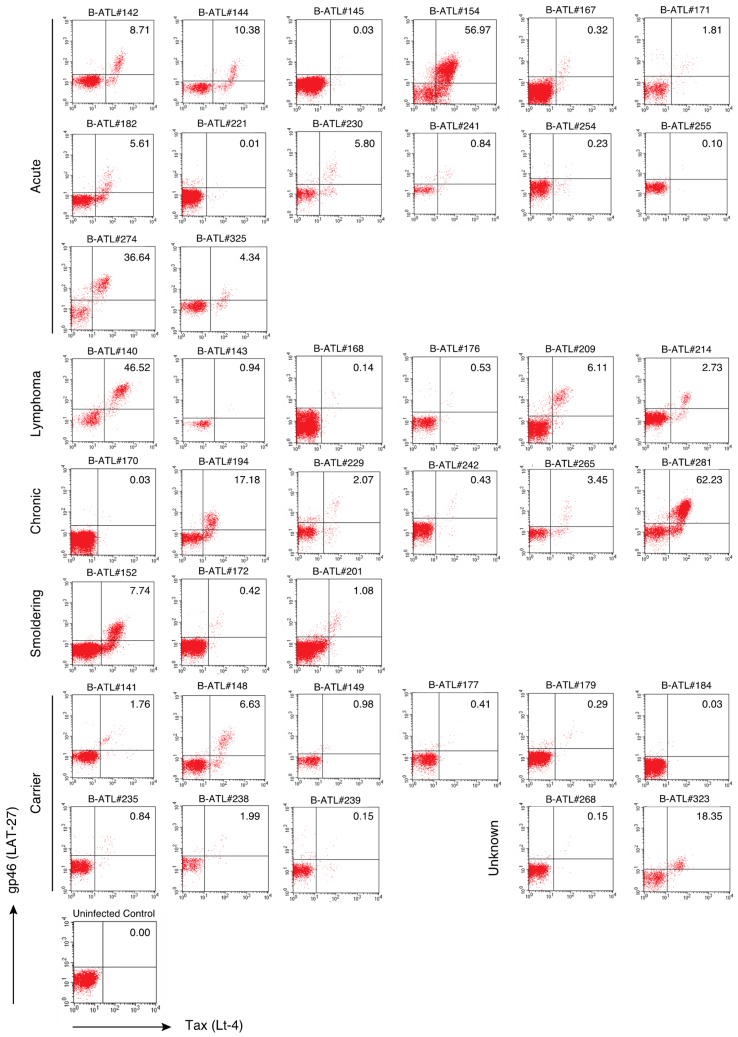
Presence of LAT-27-responsive cells in HTLV-1-infected individuals. Fresh PBMCs and LN cells from acute, lymphoma, chronic, and smoldering ATL patients and from asymptomatic carriers were cultured in IL-2-containing media for 18 h in vitro. The cells were stained with FITC-conjugated anti-gp46 antibody (LAT-27) and HyLite Fluor 647-labeled anti-Tax antibody (Lt-4). Each FCM experiment was conducted once. The number in the dot-plot shows the percentage of double positive cells for LAT-27 and Lt-4 staining.

We further investigated the conservation of the LAT-27 epitope among HTLV-1 produced by primary PBMCs and lymph node (LN) cells from patients with various subtypes of ATL or from asymptomatic carriers (Table 2) using FCM. As the level of HTLV-1 expression was suppressed in vivo [28,29,30], PBMCs and LN cells from HTLV-1-infected individuals were pre-cultured in interleukin (IL)-2-containing media for 18 h in vitro. The expression levels of the HTLV-1 antigens, gp46 and Tax, were then evaluated using LAT-27 and Lt-4 mAbs, respectively. As shown in Figure 3, although there were individual differences, gp46-expressing cells were detected in 40 HTLV-1-infected clinical samples that were positive for the Tax antigen. The percentage of gp46- and Tax-expressing cells was low in several cases (Figure 3), which suggests to some extent that these samples contained only a small number of HTLV-1-infected cells, or that HTLV-1-specific CTL might have led to the killing of infected CD4^+^ T-cells during the 18 h of cultivation [31]. There were no correlations between diagnosis and the expression levels of gp46 and Tax. Altogether, these results support that the LAT-27 epitope is well conserved in HTLV-1-infected individuals living in Okinawa.

### 2.4. hu-LAT-27 Cross-Reacts with a Peptide Carrying a P192S Substitution in the LAT-27 Epitope

**Figure 4:**
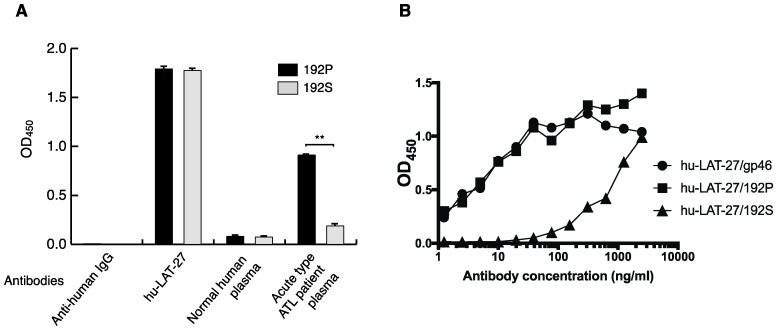
Binding of hu-LAT-27 to the synthetic peptide with substitution. (**A**,**B**) HTLV-1 gp46 synthetic peptides, with and without an amino acid substitution at position 192 (proline to serine), 192P, and 192S, respectively (see Materials and Methods), as well as a native gp46 antigen were coated onto 96-well ELISA plates and reacted with hu-LAT-27 (10 μg/mL), normal human plasma (1:400), or acute type ATL patient plasma (1:400). The binding of human IgG was probed using goat anti-human IgG-HRP. Values are shown as the means of the triplicate experiments ± SE. * *p* < 0.01 (A).

An amino acid substitution from proline to serine at position 192 (P192S) in the LAT-27 epitope has been reported in Caribbean, French Guiana, Gabon, and Brazil isolates [23,24,25]. To test whether hu-LAT-27 can react with the P192S-containing epitope, we performed an enzyme-linked immunosorbent assay (ELISA) using two synthetic gp46 peptides containing the wild-type (192P) or its substitution (192S), as well as a native gp46 antigen. An adequate amount of hu-LAT-27 could react with both the 192P and 192S peptides (Figure 4A). The plasma from an acute type ATL patient in Okinawa, which showed the highest neutralization activity against HTLV-1 in our library, responded to 192P more strongly than to the 192S peptide (Figure 4A). hu-LAT-27 could react with 192P peptide as well as with native gp46 antigen (Figure 4B). The binding affinity of hu-LAT-27 for the 192S substitution peptide was significantly lower than the affinities for the 192P peptide and native gp46 (Figure 4B). These results imply that hu-LAT-27 can also recognize HTLV-1 isolates with the P192S substitution. It remains to be tested whether hu-LAT-27 neutralize these HTLV-1 isolates.

## 3. Discussion

In this study, we demonstrated that the prototype of the gp46 amino acid sequence from position 191 to 196, namely Leu-Pro-His-Ser-Asn-Leu, in strain MT-2, which is recognized by LAT-27, is highly conserved in the current Okinawan clinical HTLV-1 isolates. Accordingly, all the clinical HTLV-1 strains tested were neutralized in vitro by hu-LAT-27, as shown by a syncytium formation assay. Furthermore, LAT-27 staining was detected in primary cultured PBMCs from patients with various subtypes of ATL and asymptomatic carriers. Notably, hu-LAT-27 could also bind to a synthetic gp46 peptide containing an amino acid substitution from proline to serine. Collectively, these results suggest that hu-LAT-27 may be effective in preventing the horizontal transmission of a broad range of HTLV-1 strains in vivo. 

The conservation of the LAT-27 neutralization epitope comprising six amino acid residues of gp46 has been reported for strains in other endemic areas, such as Romania, Zaire, Liberia, Melanesia, Australia, and Bellona [23]. On the other hand, a few isolates, including the 1010 and HS35 strains isolated from a British patient of Caribbean origin, showed P192S substitution in the neutralization epitope [23,24]. In addition, a recent study showed a high frequency of the P192S substitution in HTLV-1 strains isolated in Belem in Para, Brazil [25]. HTLV-1 strains are genetically subdivided into four major geographic subtypes: Cosmopolitan subtype A, Central African subtype B, Australo-Melanesian subtype C, and Central African/Pygmies subtype D [1]. Although Australo-Melanesian subtype C, which exhibited greater genetic diversity compared to the other subtypes, shows three nucleic acid changes in the LAT-27 epitope region, these mutations do not result in amino acid substitutions [23]. Cosmopolitan subtype A is the most prevalent type worldwide, and comprises four phylogenetic subgroups: Transcontinental, Japanese, West African, and North African [1]; only the Japanese and Transcontinental subgroups are distributed in Okinawa prefecture [32]. Phylogenetic analysis demonstrated that the 1010 and HS35 HTLV-1 strains with the P192S type of epitope, isolated from the Caribbean, belong to the West African subgroup [33], in accordance with the geographical distribution of the HTLV-1 subgroups [1]. Thus, it can be emphasized that the widely distributed HTLV-1 strains possess the original LAT-27 neutralization epitope, and that the P192S type is restricted to the West African subgroup. In addition, sporadic substitutions can occasionally occur around the LAT-27 epitope, such as L190P (Strain/GenBank: A6c15/KF053907 and A5c15/KF053899), L191H (2454/Belem/2015/MF084825, 2443/Belem/2015/MF084822, 762/Ananindeua/2011/MF084795 and 285/Belem/2010/MF084788), S194Y (TUM0072/U66294), S194T (2454/Belem/2015/MF084825), S194P (A10c12/KF053947), L196P (H3c6/KF053970), D197E (H2c9/KF054030), and D197N (A8c8/KF053943). Because hu-LAT-27 could bind to synthetic peptides with the amino acid substitution equally as well as it did with the wild-type, hu-LAT-27 may inhibit the transmission of the P192S type of HTLV-1 strains. However, a confirmatory neutralization assay and the blockade of HTLV-1 entry by hu-LAT-27 using the P192S type and other subtypes of HTLV-1 strains will need to be conducted to validate this possibility.

HTLV-1 shows remarkable homogeneity across subtypes, as its genetic diversity is known to be significantly lower than that of another RNA virus, human immunodeficiency virus type 1 (HIV-1). HIV-1 progressively expands and acquires genetic mutations in the env gene due to the low fidelity of its reverse transcriptase, which causes genetic errors during the reverse transcription of the viral genome [34]. Although the mutation rate of HTLV-1 is fourfold lower than that of HIV-1, the reverse transcriptase of HTLV-1 also has mutagenicity [35]. Indeed, it has been shown that the receptor binding region of HTLV-1 env localized outside of the LAT-27 epitope shows intra-host variability within HTLV-1-infected individuals [36]. Thus, as the LAT-27 neutralization epitope is well conserved across HTLV-1 subtypes, this region could represent a crucial target of passive immunization.

The importance of the conservation of the env gene is demonstrated using its mutants, which revealed comprehensively inserted amino acids [37]. The env gene encodes a 488-amino-acid precursor protein, gp62, that is cleaved to generate gp46 and gp21 [11]. The introduction of various insertion mutations in the env gene resulted in a complete or partial loss of gp62 cleavability. As env gene mutants cannot form a functional envelope molecule on the virus surface, the conservation of the env gene may be involved in the fitness of HTLV-1.

In conclusion, the present results show that the HTLV-1 neutralization epitope of gp46 recognized by hu-LAT-27 is widely conserved in Okinawan clinical HTLV-1 isolates. In addition to its key function in the neutralization of clinical isolates, hu-LAT-27 also appears to mediate the Fc receptor-dependent elimination of HTLV-1-infected cells in vitro through ADCC (Supplementary Figure S1). Our results indicate the potential of hu-LAT-27 as a strong candidate drug to serve as a prophylactic agent against horizontal and vertical HTLV-1 infection and possibly as a therapeutic agent against HTLV-1-related disease.

## 4. Materials and Methods

### 4.1. Cells and Cell Culture

HTLV-1-infected cell lines were generated from PBMCs and LN cells obtained from HTLV-1-infected individuals living in Okinawa prefecture, Japan. These cells were obtained with the approval from the Internal Review Committee of the University of the Ryukyus (permit no. 478 and 319-5). Written informed consent was obtained from the participants before their enrollment in this study. Details on the clinical status of the blood donors, including the diagnosis and ATL subtypes classified according to Shimoyama’s criteria [38], are provided in Table 1 and Table 2. The integration of the HTLV-1 provirus genome was confirmed by Southern blot analysis. PBMCs and LN cells were isolated by density-gradient centrifugation using Lymphocyte Separation Solution (Nacalai Tesque, Kyoto, Japan) and were cultured in RPMI-1640 medium containing 10% fetal calf serum (FCS), 100 U/mL of penicillin, 100 μg/mL of streptomycin, and 20 U/mL of IL-2. The IL-2-dependent HTLV-1-infected cell lines generated directly from HTLV-1-infected individuals in our laboratory were named 022i, 026i, 040i, 056i, 064i, 079i, 085i, 119i, 156i, 186i, 224i, 243i, 251i, 277i, 299i, and 307i. A human acute lymphocytic leukemia T-cell line, CEM, and an IL-2-independent T-cell line derived from a chronic type ATL patient (026m) were maintained in RPMI-1640 medium containing 10% FCS without IL-2. To stimulate the expression of the HTLV-1 antigens, the cells were cultured with 1 μg/mL of PGE2 (Sigma, St. Louis, MO, USA) for 24 h.

### 4.2. Antibodies and FCM

Rat anti-gp46 (LAT-27) and mouse anti-Tax (Lt-4) mAbs were established and purified in our laboratory [19,39]. These mAbs were labeled with fluorescein isothiocyanate (FITC) or HyLite Fluor 647 using commercial labeling kits (DOJINDO Molecular Technologies, Kumamoto, Japan). The hu-LAT-27 antibody was used for the HTLV-1 neutralization assay [20]. The expression of HTLV-1 gp46 and Tax antigens was detected by FCM as reported elsewhere [40,41]. Briefly, the cells were fixed with 1% paraformaldehyde for 10 min at room temperature and washed in saponin buffer (phosphate-buffered saline (PBS) containing 1% bovine serum albumin (BSA), 0.5% saponin, and 0.1% sodium azide). The cells were then resuspended in saponin buffer, stained with LAT-27 and Lt-4 for 30 min on ice, and analyzed with FACSCalibur using CellQuest software (BD Biosciences, San Jose, CA, USA). To detect the gp46- and Tax-expressing cells in HTLV-1-infected individuals, primary PBMCs and LN cells (not the cryopreserved ones) were cultured in IL-2-containing medium at a cell concentration of 2 × 10^6^ cells/mL for 18 h, followed by staining with LAT-27 and Lt-4. ATL and the carrier samples, which were comprised of over 0.01% Tax-expressing cells, were selected for analysis, as shown in Table 2 and Figure 3.

### 4.3. Genomic DNA Isolation and Sequencing

Genomic DNA was extracted using the QIAamp DNA Mini Kit (Qiagen, Hilden, Germany) according to the manufacturer’s protocol. Isolated DNA was subjected to PCR with the following set of primers: forward, 5′-tctagtcgacgctccaggat-3′, and reverse, 5′-gcaagtataattagtgctttgtagg-3′. DNA fragments were cloned into pGEM-T Easy Vector System I (Promega) and three clones were sequenced per genomic DNA specimen using the SP6 primer. The HTLV-1 gp46 gene of the MT-2 isolate was used as a reference sequence (GenBank: J02029.1).

### 4.4. HTLV-1 Neutralization Assay

HTLV-1-infected cell lines established from Okinawa inhabitants were pretreated with PGE2 for 24 h. These cells were then co-cultured with the same number of CEM cells in 24-well microplates in the presence or absence of 50 μg/mL of hu-LAT-27. After 24 h of cultivation, syncytium formation was microscopically observed using an inverted microscope at a magnification of 100×, as reported previously [19].

### 4.5. ELISA

ELISA was performed as described previously [19]. Two HTLV-1 gp46 synthetic peptides, spanning the amino acid region from position 185 to 204 (192P) and from position 185 to 200, containing a substitution from proline to serine at 192 (192S) and purified-gp46 [22] were used for this assay. These peptides were coated onto 96-well ELISA plates (Thermo Fisher Scientific, Waltham, MA, USA) with 50 μL/well (10 μg/mL) in PBS and left for 1 h at room temperature. After blocking with 1% gelatin for 10 min, the plates were washed and reacted with hu-LAT-27 (10 μg/mL), normal human plasma (1:400 dilution), or acute type ATL patient plasma (1:400 dilution), which showed the highest neutralization activity for HTLV-1 in our library (not included in Table 1 and Table 2), for 1 h at room temperature. The binding of human IgG was probed using goat anti-human IgG-HRP (Jackson ImmunoResearch Laboratories, Inc., West Grove, PA, USA). 

### 4.6. Statistics Analysis

A paired *t*-test was performed for statistical analysis. Values of *p* < 0.01 were considered statistically significant.

## Figures and Tables

**Table 1:** Establishment of HTLV-1-infected cell lines from patients with various subtypes of ATL.

	Bank Number	Cell Line	Disease Status	Age	Sex
**1**	B-ATL#022	022i	Chronic	46	M
**2**	B-ATL#026	026i	Acute	67	M
**3**	B-ATL#040	040i	Acute	37	M
**4**	B-ATL#056	056i	Acute	63	M
**5**	B-ATL#064	064i	Smoldering	51	F
**6**	B-ATL#079	079i	Chronic	64	M
**7**	B-ATL#085	085i	Acute	53	F
**8**	B-ATL#119	119i	Acute	63	F
**9**	B-ATL#156	156i	Lymphoma	77	M
**10**	B-ATL#186	186i	Chronic or Acute	54	F
**11**	B-ATL#224	224i	Acute	80	M
**12**	B-ATL#243	243i	Acute	82	M
**13**	B-ATL#251	251i	Acute	64	F
**14**	B-ATL#277	277i	Acute	73	F
**15**	B-ATL#299	299i	Acute	75	M
**16**	B-ATL#307	307i	Unknown	64	F
**17**	B-ATL#004		Acute	44	M
**18**	B-ATL#029		Acute	64	F
**19**	B-ATL#060		Acute	83	F
**20**	B-ATL#063		Acute	74	F

**Table 2:** Clinical characteristics of HTLV-1-infected individuals.

	Bank Number	Disease Status	Age	Sex		Bank Number	Disease Status	Age	Sex
**1**	B-ATL#140	Lymphoma	86	M	**21**	B-ATL#194	Chronic	73	M
**2**	B-ATL#141	Carrier	67	M	**22**	B-ATL#201	Smoldering or Pre-ATL	24	M
**3**	B-ATL#142	Acute	67	M	**23**	B-ATL#209	Lymphoma	66	F
**4**	B-ATL#143	Lymphoma	43	M	**24**	B-ATL#214	Lymphoma	68	F
**5**	B-ATL#144	Acute	66	M	**25**	B-ATL#221	Acute	72	F
**6**	B-ATL#145	Acute	64	F	**26**	B-ATL#229	Chronic	69	M
**7**	B-ATL#148	Carrier	62	F	**27**	B-ATL#230	Acute	83	F
**8**	B-ATL#149	Carrier	66	M	**28**	B-ATL#235	Carrier or Pre-ATL	67	M
**9**	B-ATL#152	Carrier or Smoldering	63	F	**29**	B-ATL#238	Carrier	65	F
**10**	B-ATL#154	Acute	71	M	**30**	B-ATL#239	Carrier	74	F
**11**	B-ATL#167	Acute	70	M	**31**	B-ATL#241	Acute	62	M
**12**	B-ATL#168	Lymphoma	68	M	**32**	B-ATL#242	Chronic	75	F
**13**	B-ATL#170	Chronic	60	M	**33**	B-ATL#254	Acute	82	M
**14**	B-ATL#171	Acute	71	F	**34**	B-ATL#255	Acute	82	F
**15**	B-ATL#172	Chronic or Smoldering	74	M	**35**	B-ATL#265	Chronic	61	M
**16**	B-ATL#176	Lymphoma	64	M	**36**	B-ATL#268	Unknown	79	F
**17**	B-ATL#177	Carrier	56	F	**37**	B-ATL#274	Acute	62	M
**18**	B-ATL#179	Carrier	61	F	**38**	B-ATL#281	Chronic	84	F
**19**	B-ATL#182	Acute	66	M	**39**	B-ATL#323	Unknown	77	F
**20**	B-ATL#184	Carrier	38	F	**40**	B-ATL#325	Acute	71	M

## Supplementary Materials

The following are available online at https://www.mdpi.com/2076-0817/9/2/82/s1. Figure S1: ADCC mediated by hu-LAT-27 mAb.
