# Supplementary material for: Conservation of a Neutralization Epitope of Human T-cell Leukemia Virus Type 1 (HTLV-1) among Currently Endemic Clinical Isolates in Okinawa, Japan
Source: Pathogens. 2020 Jan 27;9(2):82. doi: 10.3390/pathogens9020082 (PMC7168584; doi:10.3390/pathogens9020082)
Supplement: Supplementary file 1 [file pathogens-09-00082-s001.pdf]

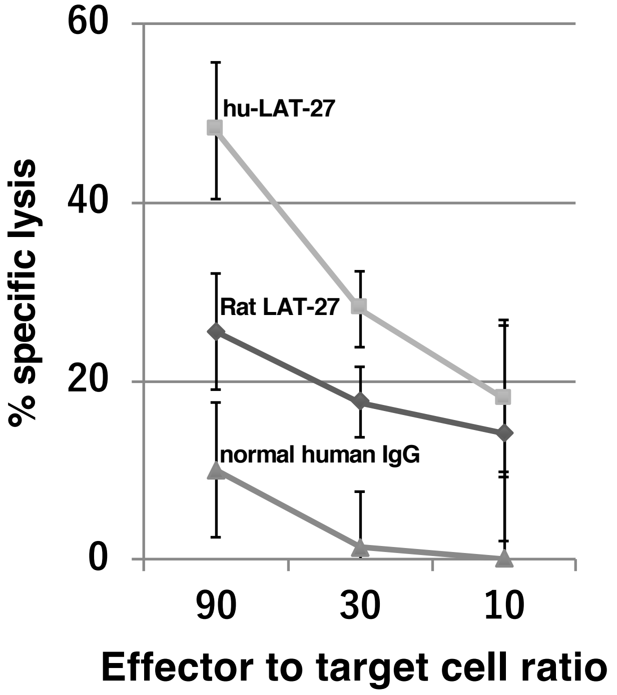

Supplemental Figure 1. ADCC mediated by hu-LAT-27 mAb.
^{51}Cr-labeled HTLV-1-infected target cells were co-cultivated with normal human PBMCs at different effector to target ratios in the presence of 5 μg/mL antibody for 24 h, and the percentage of specific lysis was calculated as previously reported (Reference 22 in the main text).
